# Multiple habitat use by declining migratory birds necessitates joined‐up conservation

**DOI:** 10.1002/ece3.4895

**Published:** 2019-02-18

**Authors:** Micha V. Jackson, Luis R. Carrasco, Chi‐Yeung Choi, Jing Li, Zhijun Ma, David S. Melville, Tong Mu, He‐Bo Peng, Bradley K. Woodworth, Ziyou Yang, Lin Zhang, Richard A. Fuller

**Affiliations:** ^1^ School of Biological Sciences University of Queensland St Lucia Queensland Australia; ^2^ Department of Biological Sciences National University of Singapore Singapore; ^3^ Spoon‐billed Sandpiper (Shanghai) Environment Protection Technology Co. Ltd Shanghai China; ^4^ Ministry of Education Key Laboratory for Biodiversity Science and Ecological Engineering, Coastal Ecosystems Research Station of the Yangtze River Estuary, and Shanghai Institute of Eco‐Chongming Fudan University Shanghai China; ^5^ Global Flyway Network Nelson New Zealand; ^6^ Department of Ecology and Evolutionary Biology Princeton University Princeton New Jersey USA; ^7^ NIOZ Royal Netherlands Institute for Sea Research Department of Coastal Systems and Utrecht University Den Burg, Texel The Netherlands; ^8^ Conservation Ecology Group, Groningen Institute for Evolutionary Life Sciences University of Groningen Groningen The Netherlands; ^9^ Present address: School of Environmental Science and Engineering Southern University of Science and Technology Shenzhen China

**Keywords:** aquaculture, China, coastal land use, land claim, shorebirds, stopover ecology, working coastal wetlands

## Abstract

Many species depend on multiple habitats at different points in space and time. Their effective conservation requires an understanding of how and when each habitat is used, coupled with adequate protection. Migratory shorebirds use intertidal and supratidal wetlands, both of which are affected by coastal landscape change. Yet the extent to which shorebirds use artificial supratidal habitats, particularly at highly developed stopover sites, remains poorly understood leading to potential deficiencies in habitat management. We surveyed shorebirds on their southward migration in southern Jiangsu, a critical stopover region in the East Asian Australasian Flyway (EAAF), to measure their use of artificial supratidal habitats and assess linkages between intertidal and supratidal habitat use. To inform management, we examined how biophysical features influenced occupancy of supratidal habitats, and whether these habitats were used for roosting or foraging. We found that shorebirds at four of five sites were limited to artificial supratidal habitats at high tide for ~11–25 days per month because natural intertidal flats were completely covered by seawater. Within the supratidal landscape, at least 37 shorebird species aggregated on artificial wetlands, and shorebirds were more abundant on larger ponds with less water cover, less vegetation, at least one unvegetated bund, and fewer built structures nearby. Artificial supratidal habitats were rarely used for foraging and rarely occupied when intertidal flats were available, underscoring the complementarity between supratidal roosting habitat and intertidal foraging habitat. Joined‐up artificial supratidal management and natural intertidal habitat conservation are clearly required at our study site given the simultaneous dependence by over 35,000 migrating shorebirds on both habitats. Guided by observed patterns of habitat use, there is a clear opportunity to improve habitat condition by working with local land custodians to consider shorebird habitat requirements when managing supratidal ponds. This approach is likely applicable to shorebird sites throughout the EAAF.

## 1 INTRODUCTION

Long‐distance migratory birds, like all migratory species, depend on multiple habitats at different points in space and time. Consequently, a reduction in the quality of one habitat used can have far‐reaching consequences for a species, even if its other habitat(s) remain in good condition. For example, the annual survival of Red Knot *Calidris canutus rufa* in North America is linked to the spawning abundance of horseshoe crabs at the midpoint of their annual migration (McGowan et al., 2011) and female American Redstarts *Setophaga ruticilla *that occupy high‐quality nonbreeding habitat in Central and South America produce more young on their breeding grounds in Canada (Norris et al., 2004). Successful conservation of migratory species therefore requires adequate protection across large‐scale habitat requirements. Yet formal habitat protection often fails to meet this requirement, with less than 10% of migratory birds adequately protected across their life cycle, compared with nearly half of sedentary species (Runge et al., 2015).

Many bird species also have multiple habitat requirements on much smaller spatiotemporal scales. Habitat switching may be diurnal, such as for owls that roost in forests during the day and forage in grasslands at night (Framis, Holroyd, & Mañosa, 2011). Coastal species may require different habitats over the course of the tidal cycle, as with breeding Black‐headed Gulls *Larus ridibundus *that switch between terrestrial and marine feeding sites based on prey availability linked with tide state (Schwemmer & Garthe, 2008).

Migratory shorebirds of the East Asian Australasian Flyway (EAAF) are an imperilled group of species that use multiple habitats across both large and small spatiotemporal scales.

At the scale of the annual cycle, migratory shorebirds travel enormous distances between breeding grounds in the arctic/subarctic, where they occupy open tundra and meadows, and nonbreeding grounds near the equator and into the southern hemisphere, where they occupy coastal and inland wetlands (Conklin, Verkuil, & Smith, 2014). At stopover and staging sites in between, wetlands with high productivity provide critical feeding and resting habitat necessary to complete migration successfully (Ma et al., 2013). In the EAAF, the scale and rate of intertidal habitat loss and degradation in Yellow Sea staging areas (Melville, Chen, & Ma, 2016; Murray, Clemens, Phinn, Possingham, & Fuller, 2014) are well accepted as the primary driver of severe population declines in multiple shorebird species (Amano, Székely, Koyama, Amano, & Sutherland, 2010; Piersma et al., 2016 Studds et al., 2017). This conservation crisis has prompted a focussed research effort to highlight negative consequences of coastal development and armouring on migratory waterbirds and the need to halt intertidal habitat loss (Choi et al., 2018; Ma et al., 2014; Murray, Ma, & Fuller, 2015; Piersma et al., 2017; Yang et al., 2011).

Despite the focus on intertidal habitat conservation, at a relatively small scale on nonbreeding grounds (including staging and stopover sites), shorebirds regularly switch between intertidal habitat, generally used for foraging at lower tides, and supratidal habitat, often used for high tide roosting—an important period of sleep, rest, and digestion (Choi et al., 2014; Rogers, 2003). In the Yellow Sea and elsewhere in the EAAF, supratidal habitats are also used by some shorebirds for foraging (e.g., Masero et al., 2000; Green, Sripanomyom, Giam, & Wilcove, 2015; Lei et al., 2018). The same coastal development that has contributed to intertidal flats loss in the Yellow Sea has also caused most natural supratidal wetlands to be replaced by artificial “working wetlands” including aquaculture, agriculture, and salt production (Cai, van Vliet, Verburg, & Pu, 2017; Xu, Gao, & Ning, 2016), and shorebirds are known to utilize such artificial habitats as they do natural supratidal wetlands (Basso, Fonseca, Drever, & Navedo, 2017; Masero & Pérez‐Hurtado, 2001). Yet relatively little attention has been given in the EAAF to how coastal development affects the complementarity between intertidal and supratidal habitats for shorebirds at a site level, or the management that artificial supratidal wetlands created or modified by the land claim process may require to prevent further shorebird population declines.

Here, we evaluate the importance of artificial supratidal habitats and the relationship between intertidal and supratidal habitats for migratory shorebirds in Rudong, Jiangsu province, China, one of the most important stopover sites in the EAAF (Peng et al., 2017). We quantify shorebird abundance on artificial supratidal habitats and estimate how often inundation of intertidal habitat necessitates movement into the supratidal zone. To inform management needs, we determine which biophysical features of artificial supratidal habitats are associated with shorebird abundance, and identify whether artificial supratidal habitats are used for foraging, roosting, or both. We conclude by exploring potential approaches to implementing supratidal habitat management in Rudong for the benefit of migratory shorebirds, and the applicability of our results to other sites.

## 2 MATERIALS AND METHODS

### 2.1 Study area

The coast around Rudong in southern Jiangsu province, eastern China, is one of the most important stopover regions for migratory shorebirds in the EAAF (Bai et al., 2015; Peng et al., 2017; Conklin et al., 2014) with some of the widest remaining intertidal flats on China's coast (Wang, Zhu, & Wu, 2002). More than 100,000 shorebirds occur here during migration including 20 species in internationally important numbers (Ramsar Convention Criteria 6, >1% of the total flyway population) during southward migration (Bai et al., 2015; Peng et al., 2017). It is the most important known migration stopover site for the Critically Endangered Spoon‐billed Sandpiper *Calidris pygmaea*, with 225 individuals recorded in 2014 (Peng et al., 2017) of an estimated global population of <250 breeding pairs (Clark et al., 2016). It is also the most important known migration stopover site for the Endangered Nordmann's Greenshank *Tringa guttifer*, with 1,110 individuals recorded in 2015 (Bai et al., 2015; Peng et al., 2017), equal to almost the entire estimated global population (Conklin et al., 2014; Zöckler, Li, Chowdhury, Iqbal, & Chenxing, 2018).

Most intertidal flats along the Rudong coastline have been partially enclosed for land claim (i.e., upper parts of the flats have been claimed but some intertidal areas lower down the shore remain; Zhang et al., 2011; Piersma et al., 2017), and most of the shoreline is now formed by a concrete seawall. Almost no natural wetlands remain inside the seawall, with aquaculture, agriculture, and urban and industrial infrastructure dominating land use (Cai et al., 2017). Therefore, if seawater reaches the seawall at high tide thereby covering remaining intertidal flats, generally only “artificial” supratidal habitat (i.e., habitat occurring as a result of planned construction activities that have deliberately converted natural intertidal flats into artificial nontidal land) will be available for shorebirds. The limited availability of supratidal roosting sites is a threat to shorebirds in the Rudong region (Peng et al., 2017), but little detailed information on supratidal habitat use is currently available.

### 2.2 Shorebird surveys

**Figure 1:**
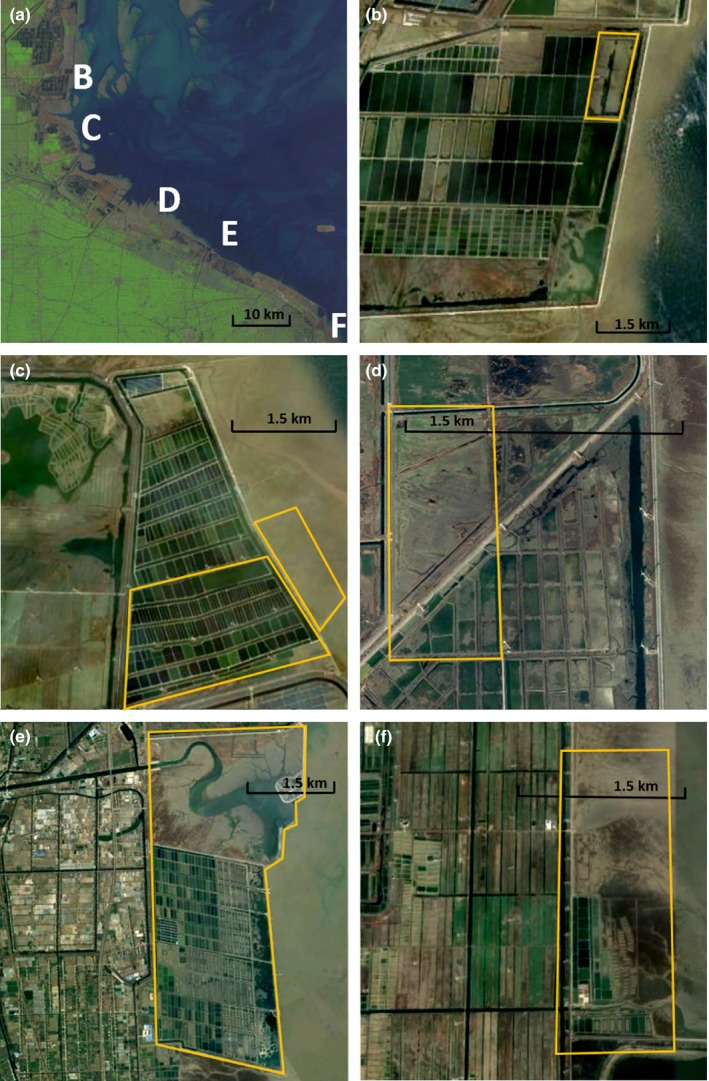
Satellite images of count regions (Panel A Landsat, panels B–F Google Earth). Panel A shows the whole study area with letters B–F demarking survey regions that correspond to detailed images in panels B–F (rotated so that intertidal flats always appear on the right‐hand side of the image). Panel B: Dongtai undeveloped pond outlined and surveyed from the seawall. Panel C: Hai'an intertidal flats and aquaculture complex; intertidal flats and 21 randomly selected ponds stratified by distance from intertidal flats and size within the outline were surveyed. Panel D: Fengli aquaculture complex; wet ponds of varying sizes and larger dry ponds are intersected by a road; all ponds outlined (10 wet, one dry) were surveyed. Panel E: Ju Zhen undeveloped pond and aquaculture complex; undeveloped pond and 18 randomly selected ponds stratified by distance from intertidal flats and size within the outline were surveyed. Panel F: Dongling; ~1 km strip of intertidal flats were surveyed; aquaculture ponds within the outline were checked but no shorebirds were observed

We conducted surveys from August to October 2017, covering the peak southward migration period for shorebirds. We established five survey sites along ~75 km of coastline in Dongtai, Hai'an, and Rudong counties at intertidal and supratidal aggregation points identified during surveys in May 2017 (Zhang & Laber, 2017) and a 3‐day scoping trip in July 2017. From north to south, we counted shorebirds at Dongtai (supratidal undeveloped pond; Figure 1b), Hai'an (intertidal flats roost and supratidal aquaculture ponds; Figure 1c), Fengli (supratidal aquaculture ponds; Figure 1d), Ju Zhen (supratidal undeveloped pond and aquaculture ponds; Figure 1e), and Dongling (intertidal flats roost and aquaculture ponds; Figure 1f). At Hai'an and Ju Zhen where we were able to systematically survey multiple aquaculture ponds, individual ponds were randomly selected from large aquaculture complexes (*n* = 21 ponds at Hai'an and *n* = 18 ponds at Ju Zhen) and stratified by distance from intertidal flats (<1 and 1–2 km from intertidal flats) and size (<3 and >5 ha). At Fengli, all adjacent ponds (*n* = 11) of varying sizes within a subsection of an aquaculture complex were surveyed; a more detailed description of surveys sites is in Supporting Information S1.

To quantify their use as roosting sites, we counted shorebirds on artificial supratidal habitats within 3 hr on either side of high tide. Because we expected birds to enter supratidal habitats when intertidal flats became covered with seawater, we recorded the state of adjacent intertidal flats during the survey as either covered (seawater had reached the seawall) or uncovered (seawater had not reached the seawall). We varied the timing of counts to provide an estimate of the minimum high tide height (China National Marine Data & Information Service, 2016) at which intertidal flats became covered (full count schedule in Supporting Information S2). Because the undeveloped ponds at Dongtai and Ju Zhen were directly adjacent to the seawall facilitating easy access during surveys, here we estimated how long intertidal flats were covered during high tide (measured as the time from when seawater first reached the seawall to when the first intertidal flats became exposed on the falling tide) to indicate how long shorebirds were without foraging opportunities on adjacent intertidal flats.

To get an idea of shorebird numbers within the aquaculture complexes, we calculated a mean total aquaculture area count (counts were conducted across 1–2 days) at Hai'an, Fengli, and Ju Zhen using the maximum count for any ponds that were counted multiple times in the count period. It should be noted, however, that only a random sample of ponds from within these aquaculture complexes was surveyed so the total number of birds within the complex is expected to have been higher than our total aquaculture area counts.

We identified migratory shorebirds to species level or as curlew sp. (i.e., Far Eastern Curlew *Numenius madagascariensis* or Eurasian Curlew *N. arquata orientalis*), godwit sp. (i.e., Bar‐tailed Godwit *Limosa lapponica* or Black‐tailed Godwit *L. limosa*), Sand Plover sp. (i.e., Greater Sand Plover *Charadrius leschenaultii* or Lesser Sand Plover *C. mongolus*), or unidentified small/medium shorebird when species‐level identification was not possible.

### 2.3 Factors affecting roost site choice

Shorebirds choose roost sites that minimize predation risk, disturbance, and the energetic costs associated with travel distance from foraging grounds (Jackson, 2017; Luis, Goss‐Custard, & Moreira, 2001; Rogers, 2003). To minimize predation risk, shorebirds tend to avoid tall vegetation or built structures, favoring good visibility around the roost (Rogers, Piersma, & Hassell, 2006; Zharikov & Milton, 2009). Water level also influences occupancy and foraging opportunities, with different species preferring different depths (Rogers, Stamation, Loyn, & Menkhorst, 2015) and some species roosting away from water altogether. We therefore recorded for each artificial supratidal pond its distance to the seawall; water cover; vegetation cover; the number of unvegetated bunds (bund meaning the banks surrounding the pond, sometimes called berms) around the pond (0–4 for each rectangular pond); the number of structures in the vicinity of the pond; and pond size as possible biophysical variables affecting roost choice (Table 1).

**Table 1:** Biophysical survey variables

Variable	Description
Intertidal flats cover	1 = seawater was against the seawall during the count 0 = seawater did not reach the seawall during the count
Water cover (%)	It was not feasible to measure water depth throughout the pond so we estimated the percentage cover of water over the surface area of the whole pond
Distance (km)	Distance to seawall measured in kilometers using Google Earth
Vegetation cover (%)	Estimated nonwater surface area covered by vegetation, measured as <10%, 10%–30%, 30%–50%, 50%–70%, or >70%
Bund	Number of unvegetated bunds (i.e., the bank surrounding the pond, sometimes called berms) for each pond, recorded as 0–4, represented in the model as 1 = at least one unvegetated bund; 0 = no unvegetated bunds
Structures	Number of structures (telephone/electricity poles/wires, buildings and trees) within 10 m of the perimeter of the pond
Size	Pond size measured in hectares using Google Earth

We modeled total shorebird abundance on artificial supratidal habitats in relation to biophysical variables using generalized linear mixed‐effects models. Each model included random intercepts for survey region (Hai'an, Fengli, or Ju Zhen) and pond identifier to account for repeated counts of total abundance within ponds and within regions in our survey design. The undeveloped pond at Dongtai was excluded because access and logistical constraints meant that other ponds in Dongtai were not incorporated into a robust survey design in a comparable way to other regions (i.e., ponds randomly selected and stratified by size and distance). Prior to model fitting, we checked for multicollinearity among explanatory variables; all had variance inflation factors <1.4 in a linear model. Variables were scaled to *z* scores by subtracting the mean and dividing by standard deviation. Models were fitted using the glmmTMB package implemented in Rv3.5.0 (R Core Team, 2016) because it enables straightforward comparison of model distributions appropriate for animal counts, including zero‐inflated mixed models (Brooks et al., 2017).

We first modeled the null and full models using a Poisson distribution; however, by calculating the sum of squared Pearson residuals and comparing it to the residual degrees of freedom, we identified overdispersion problems with selecting a Poisson distribution. A negative binomial distribution was instead selected to correct for overdispersion. We then conducted model selection using an information theoretic approach (AICc: Burnham & Anderson, 2001) on eight candidate models that combined variables we hypothesized would be highly important (intertidal flats cover and water cover), moderately important (vegetation cover, presence of an unvegetated bund, and an interaction term between the two), and less important (pond size, distance, and structures) for explaining variation in shorebird abundance. We used the R package DHARMa to check deviation of quantile residuals of the most supported model from expected values (Hartig, 2018).

### 2.4 Ecological function of supratidal habitats

Supratidal habitats can serve different ecological functions for shorebirds including roosting habitat, supplemental foraging habitat, and/or preferred foraging habitat (Dias, Lecoq, Moniz, & Rabaca, 2013; Masero et al., 2000). To evaluate ecological function, we surveyed artificial supratidal ponds in each region (except Fengli) at least once when adjacent intertidal flats were exposed (i.e., seawater had not reached the seawall) to determine whether or not they were used by shorebirds when intertidal flats were available (i.e., not covered; Supporting Information S2). When time permitted, we also recorded the total number of individual birds of each species that was observed foraging (i.e., actively feeding rather than roosting or loafing) during artificial supratidal pond surveys. Foraging observations were made at the time each shorebird was counted; we did not observe the behavior of individual birds for an extended duration. If supratidal habitats are not used when intertidal flats are available and a low proportion of shorebirds are observed foraging, this suggests that supratidal habitats are used primarily as roosting sites.

## 3 RESULTS

### 3.1 Extent and frequency of supratidal habitat use

**Figure 2:**
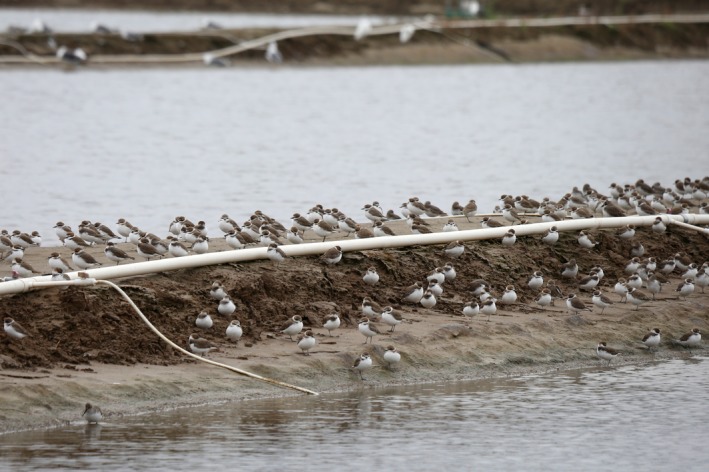
Migratory shorebirds occupying a bund between active aquaculture ponds in Hai'an, Jiangsu Province, China

By summing the maximum count of each species for each supratidal pond surveyed, we found that a minimum of 35,642; 29,562; and, 20,495 shorebirds of 37 species used artificial habitats during our count periods in August, September, and October, respectively, including internationally important numbers of Eurasian Curlew (globally Near Threatened (IUCN, 2018), max count 2,400), Spotted Redshank *Tringa erythropus *(max count 485), Nordmann's Greenshank (globally Endangered (IUCN, 2018), max count 250), Dunlin *Calidris alpina *(max count 6,500), Spoon‐billed Sandpiper (globally Critically Endangered (IUCN, 2018), max count 20), Far Eastern Oystercatcher *Haematopus [ostralegus] osculans *(globally Near Threatened (IUCN, 2018), max count 360), Grey Plover *Pluvialis squatarola* (max count 2,000), and Kentish Plover *Charadrius alexandrinus* (max count 3,181; Figure 2; Supporting Information S3). Species composition differed among sites, with small species, particularly Dunlin, Kentish Plover, and Lesser Sand Plover dominating supratidal sites except Dongtai, where large shorebirds (i.e., Eurasian Curlew, Bar‐tailed Godwit, Grey Plover, and Great Knot *Calidris tenuirostris*) comprised 30–40% of the individuals recorded (Supporting Information S3 and S7).

Mean (±*SE*) shorebird count on artificial supratidal habitats when intertidal flats were covered by seawater was as follows: Dongtai (undeveloped pond): 17,534 ± 3,351, maximum 24 species recorded; Hai'an (aquaculture): 3,355 ± 641 (mean total aquaculture area count), maximum 19 species recorded in any one pond; Fengli (aquaculture); 4,810 (total aquaculture area count; not presented as a mean because only surveyed once), maximum 10 species recorded in any one pond; Ju Zhen (undeveloped pond): 5,107 ± 862, maximum 16 species recorded; and Ju Zhen (aquaculture): 19 ± 5 (mean total aquaculture area count), maximum five species recorded in any one pond (Table 2). We did not observe shorebirds using supratidal areas at Dongling, where the mean count on the intertidal flats roost was 12,832 ± 1,322 at high tide. Mean and standard error for each individual aquaculture pond in Hai'an, Fengli, and Ju Zhen are in Supporting Information S4.

**Table 2:** Shorebird survey results from roosting sites around Rudong in autumn 2017

Region	Mean count ± *SE* (*n* counts); intertidal flats covered	Max number of species	Mean count ± *SE* (*n* counts); intertidal flats uncovered	Max number of species
Dongtai undeveloped	17,534 ± 3,351 (*n* = 3)	24	1,382 ± 619 (*n* = 5)	12
Hai'an intertidal flats roost	5,212 ± 1,046^b^ (*n* = 6)	20	5,352 c (*n* = 1)	12
Hai'an aquaculture^a^	3,355 d ± 641 (*n* = 4)	19	266 d ± 258 (*n* = 3)	6
Fengli aquaculture^a^	4,810 e (*n* = 1)	10	Not observed	N/A
Ju Zhen undeveloped	5,107 ± 862 (*n* = 3)	16	0 (*n* = 1)	0
Ju Zhen aquaculture^a^	19 d ± 5 (*n* = 3)	5	6 e (*n* = 1)	2
Dongling intertidal flats roost	N/A	N/A	12,832 ± 1,322^c^ (*n* = 3)	22

Notes

Counts (mean ± *SE*) from individual aquaculture ponds in Hai'an, Fengli, and Ju Zhen are given in Supporting Information S4.

a Total shorebird abundance within the aquaculture complex likely higher than reported counts because only a random sample of ponds from within the complex was surveyed.

b Prior to intertidal flats being covered and all birds departing.

c Birds remained on intertidal flats.

d Mean total aquaculture area count calculated using the maximum count for any ponds that were counted multiple times in one count period.

e Total aquaculture area count calculated using the maximum count for any ponds that were counted multiple times in the count period; not a mean as this area was only surveyed once.

**Figure 3:**
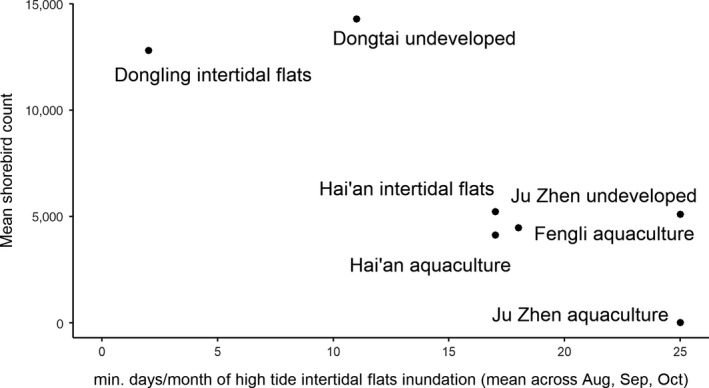
Indicative extent of artificial habitat use by shorebirds in Rudong when intertidal flats were inundated at Dongtai, Hai'an, Fengli, and Ju Zhen supratidal areas, and at high tide at Hai'an and Dongling intertidal flats

Based on the minimum tide level when we observed seawater hitting the seawall, we estimate that birds had to leave intertidal flats and enter artificial supratidal habitats on average 11 ± 0.6, 17 ± 0.3, 18 ± 0.3, 25 ± 0.3, and 2 ± 0.6 days per month at Dongtai, Hai'an, Fengli, Ju Zhen, and Dongling, respectively (Supporting Information S5). On spring high tides, intertidal flats were covered for about 1 hr at Dongtai and more than 4 hr at Ju Zhen. Given the semidiurnal nature of the tides in southern Jiangsu, this situation would occur twice daily during the spring tide period. The number of birds we counted was negatively correlated with the number of days that intertidal flats were covered at high tide (Pearson correlation coefficient = −0.84; Figure 3), suggesting birds may favor sites where intertidal flats remain accessible for longer.

### 3.2 Factors affecting roost site choice

**Figure 4:**
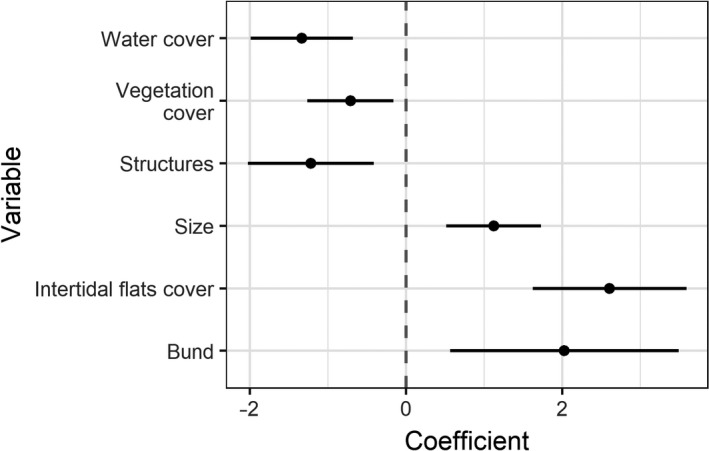
Effects of biophysical features on shorebird abundance in artificial supratidal ponds. Points show the estimated coefficients from the most supported model (Table 3) with 95% confidence intervals

The most supported model included all variables except distance to seawall (Table 3; full model output in Supporting Information S6). Shorebird counts were positively associated with intertidal flats being covered, the pond having at least one unvegetated bund, and pond size; and negatively associated with greater water cover, more extensive vegetation cover, and more structures in the vicinity of the pond (Figure 4). For the ponds studied (all ≤2 km from the seawall), distance to the seawall was not significant.

**Table 3:** Candidate models of variables influencing shorebird abundance in artificial supratidal ponds

Model	AICc	*df*	ΔAICc
*Null model: Shorebird abundance ~1 + (1 | Region) + (1 | Pond)*			
**NULL + Intertidal flats cover + Water cover + Vegetation cover + Bund + Size + Structures**	980.4	10	0.0
NULL + Intertidal flats cover + Water cover + Vegetation cover + Bund + Size + Distance + Structures	982.7	11	2.2
NULL + Intertidal flats cover + Water cover + Vegetation cover + Bund	986.7	8	6.2
NULL + Intertidal flats cover + Water cover + Vegetation cover + Bund + Vegetation cover*Bund	986.9	9	6.5
NULL + Intertidal flats cover + Water cover	989.9	6	9.4
NULL + Water cover + Vegetation cover + Bund + Size + Structures	1,001.4	9	21
NULL + Water cover	1,007.4	5	26.9
NULL + Intertidal flats cover	1,017.1	5	36.6
NULL	1,032.9	4	52.5

Note

Most supported model shown in bold. Region (Hai'an, Fengli, or Ju Zhen) and pond treated as random effects and denoted by **|**. AICc is a second‐order form of AIC adjusted for small sample sizes; *df* is degrees of freedom.

The single largest aggregation of birds occurred on the undeveloped pond at Dongtai (Table 2). In Ju Zhen, where there was both an undeveloped pond and a large aquaculture complex adjacent to intertidal flats, an average of 5,107 birds used the undeveloped pond while almost none used the aquaculture ponds (Table 2). Both of the undeveloped ponds contained some water (30%–50% water cover in Dongtai over three survey months; 40%–50% water cover in Ju Zhen over two survey months) and bare mud interspersed with vegetation (vegetation cover 10%–30%; Supporting Information S7). In contrast, water cover approached 100% in many of the aquaculture ponds in Hai'an and Ju Zhen where fewer birds were found. At Fengli, hundreds to thousands of birds used ponds with lower (<60%) water cover, while ponds with water cover approaching 100% held very few birds (Supporting Information S7). Although it was not feasible to measure water depth directly, ponds approaching 100% water cover appeared to contain water too deep for shorebirds to stand in (>20 cm depth). Water cover also affected whether birds roosted on the bunds between ponds versus within the pond itself (Supporting Information S7).

### 3.3 Ecological function of supratidal habitats

Mean total shorebird counts were much higher when intertidal flats were covered by seawater than when they were exposed in all regions except Dongling (where intertidal flats were never covered; Table 2). At low tide and at high tides when intertidal flats remained uncovered, meant count at Dongtai was <10% of the mean count when intertidal flats were covered (1,382 ± 619, *n* = 5 vs. 17,534 ± 3,351, *n* = 3), while almost no birds were observed at Hai'an or Ju Zhen when intertidal flats were uncovered (Table 2).

When intertidal flats were covered and we recorded foraging behavior, <1% of the birds at Dongtai (*n* = 1 count), 1% at Hai'an (*n* = 56 counts), ~7% at Ju Zhen (*n* = 2 counts), and ~7% at Fengli (*n* = 16 counts) were observed foraging (Supporting Information S8). However, the proportion of foraging birds differed by species; for example, at Fengli 94% of Red‐necked Stints *Calidris ruficollis*, 92% of Marsh Sandpipers *Tringa stagnatilis,* and 86% of Spoon‐billed Sandpipers were observed foraging compared with <3% of more numerous Kentish Plovers and Dunlins (Supporting Information S8).

## 4 DISCUSSION

### 4.1 Need for joined‐up conservation

It is clear that artificial supratidal habitats, particularly undeveloped ponds and aquaculture ponds, form an integral part of the daily cycle of shorebirds in Rudong during southward migration. We observed between ~20,000 and ~36,000 shorebirds using artificial habitats each month, including internationally important numbers of eight species, and believe these counts underestimated shorebird abundance because: (a) we only counted randomly selected aquaculture ponds in the Hai'an and Ju Zhen complexes; (b) we did not count Fengli in August and September or Ju Zhen in October; and (c) some shorebirds would have departed the study area before all individuals had arrived (Choi et al., 2016), meaning peak numbers observed across the period represent only part of the population that used the area. Among our survey regions, only shorebirds at Dongling were able to remain on intertidal flats throughout the tidal cycle and were only observed roosting on the seaward side of the seawall. This is consistent with the main finding of Rosa, Encarnacao, Granadeiro, and Palmeirim (2006) that given the option between roosting on the top portion of intertidal flats and artificial supratidal habitats, shorebirds will choose to remain on intertidal flats to minimize predation and disturbance risk. Yet subsequent to our fieldwork, land claim has occurred at the Dongling intertidal roost and it is now likely that these birds (averaging almost 13,000 across three monthly counts) require artificial supratidal roosts at high tide as well (L. Zhang, pers. obs.).

Widespread use of artificial supratidal habitats by migrating shorebirds in Rudong is unsurprising because the intertidal flats where they aggregate are covered by seawater during spring high tides and almost no natural supratidal habitat remains in this region following extensive land claim along the coast (Cai et al., 2017). Similar behavior has been recorded elsewhere in the EAAF, for example, in Changhua (Bai et al., 2018), the Mai Po Nature Reserve (WWF Hong Kong, 2013), Inner Gulf of Thailand (Sripanomyom, Round, Savini, Trisurat, & Gale, 2011), and mainland China (e.g. He et al., 2016).

It is nonetheless clear from our results that birds concurrently depend on natural intertidal and artificial supratidal habitats in Rudong. Few shorebirds used artificial supratidal areas when intertidal flats were not covered by seawater and most shorebirds did not appear to forage substantively in supratidal areas. This indicates that the two habitats serve different functional roles across one connected area, depending on the tide. There is therefore a management imperative to maintain both suitable artificial supratidal habitat and natural intertidal habitat, and degradation or loss of either could lead to further pressure on shorebird populations. Further research in Rudong should seek to identify precise movement patterns for individual shorebirds between intertidal feeding areas and supratidal habitats. Telemetry or mark‐resighting studies could be used to determine whether or not individual shorebirds consistently use supratidal habitats closest to their foraging areas; if this is the case, prioritizing management at supratidal sites adjacent to the largest shorebird aggregations (or target species aggregations) on intertidal flats would be effective.

### 4.2 Management of artificial supratidal habitats

Shorebirds were more abundant in ponds with less water cover, less vegetation cover, an unvegetated bund, and fewer built structures in the vicinity, consistent with previous research and predation avoidance tactics (He et al., 2016; Rogers, 2003; Zharikov & Milton, 2009). Our model also associated larger ponds with higher shorebird abundance, but pond size is perhaps less important than water and vegetation cover because we surveyed several large ponds that had high water and vegetation cover that did not support any shorebirds across the survey period. Foraging observations suggest that only those ponds with water cover significantly below 100% presented any substantive foraging opportunity (Supporting Information S8). Distance to the seawall was not included in the best‐fit model, likely because areas that we were able to survey were all within 2 km of the seawall and therefore well inside maximum observed travel distances from foraging to roosting sites for shorebirds (Jackson, 2017; Rogers, 2003). We nonetheless included this variable because if the distance between supratidal ponds and the seawall within 2 km had affected roost choice, this would be an important consideration for management; however, our results do not suggest that distance within 2 km was a significant influence on roost choice in our study area.

Several areas of additional research would help to develop more specific management strategies for the region. One limitation of our study was that only the total shorebird abundance could be modeled because there were insufficient data to model individual species or size classes. Thus, the results are primarily driven by the more common species, most of which are not of immediate significant conservation concern. Completing additional counts of target species (e.g., threatened species) and modeling their occurrence against biophysical variables could clarify whether species of interest fit the general pattern described in this study. In addition, while water cover significantly below 100% is likely preferred across most shorebird species, optimum water depth differs by species (Rogers et al., 2015) and size‐class (i.e., leg length) has been used as a predictor affecting shorebird numbers at different water levels on artificial supratidal habitats elsewhere (e.g., Green et al., 2015). Future research could usefully explore whether foraging activity at supratidal sites in Rudong is negatively related to body size, as has been documented elsewhere (e.g., Nol, MacCulloch, Pollock, & McKinnon, 2014). If smaller species are more likely than larger ones to forage during the high tide period when artificial supratidal habitats are being occupied, then managers should regulate water levels to optimum depth for shorter‐legged species. Research on disturbance levels and their possible impacts on roosting shorebirds would also be beneficial to see if otherwise optimal roosting areas are not currently being utilized because disturbance levels are too high. Lastly, a more fully randomized selection of supratidal ponds may be more desirable in a future study; however, on‐ground realities relating to access and road condition make this challenging.

Overall, we nonetheless feel confident in making a general recommendation based on our results that the maintenance of a network of ponds situated along the coastal seawall near large intertidal shorebird aggregations: (a) within at minimum 2 km of the mudflat; (b) with incomplete water cover (which would result in at least some areas of bare mud and shallow water of different depths across the pond); and (c) with minimal vegetation, would provide significant benefits to multiple species, particularly during peak migration months when energy budgets are most critical.

### 4.3 Implementing joined‐up management

Several studies have suggested partnerships with local authorities and land users as a means to provide shorebird habitat within existing working wetlands (e.g., Sripanomyom et al., 2011; Navedo, Fernández, Fonseca, & Drever, 2014). Innovative approaches to partnerships with local land users can ensure that resources are allocated efficiently and provide local benefits. For example, in California, a reverse auctioning system is used to create temporary wetlands in agriculture fields at locations and times most beneficial to migrating shorebirds (Reynolds et al., 2017). Potential strategies in Rudong could include sequential aquaculture harvesting (e.g., Navedo, Fernández, Valdivia, Drever, & Masero, 2016), paying a fee to optimize water levels for shorebirds in aquaculture ponds during peak migration periods, or management of ponds in the supratidal landscape solely for waterbird conservation by an appropriate entity. Nonetheless, significant research is required to determine the feasibility and relative efficiency of alternative strategies on a local level.

Policy developments in China suggest that loss of intertidal flats from land claim for development will slow. Several intertidal areas have been proposed as tentative sites for World Heritage listing, and a recent announcement from the Chinese government detailed that business‐related land claim is to cease and decisions on future land claim activities made only by the central government (Lei, 2018; Melville, 2018; Stokstad, 2018). Preventing further loss of intertidal flats will hopefully slow the rapid decline of many shorebird species, yet beneficial effects may be undermined unless adjacent supratidal habitats are also managed for shorebird conservation.

Migrating shorebirds almost certainly rely on artificial supratidal habitats as they do in Rudong across several regions of the EAAF due to similarity in coastal development and land use. Coastal degradation associated with economic growth is widespread across China (He et al., 2014), an estimated 75% of intertidal flats have also been lost to land claim in the Republic of Korea (Moores, Rogers, Rogers, & Hansbro, 2016), and supratidal land use patterns similar to Rudong's have been documented in areas important to shorebirds elsewhere in China (e.g., Yang et al., 2011; Xu et al., 2016; C. Choi pers. obs.) and in Thailand (e.g., Sripanomyom et al., 2011). Coastal aquaculture is very prevalent in Asia, which as a whole accounts for 89% of the world's production (by volume) with China the largest single producer (Bostock et al., 2010). Of all land claim of intertidal flats between 1977 and 2015 along the central Jiangsu coast, 43% was for aquaculture (Cai et al., 2017), and aquaculture and salt production are both prevalent in other coastal regions of China (e.g., Xu et al., 2016). A large‐scale analysis is urgently needed to quantify the overall dependence of the migratory shorebirds of the EAAF on artificial supratidal habitats and prioritize management action accordingly.

## CONFLICT OF INTEREST

None declared.

## AUTHOR CONTRIBUTIONS

M.V.J, C.‐Y.C., J.L., L.Z., and R.A.F. conceived the ideas and designed methodology; M.V.J., J.L., Z.Y., and L.Z. collected the data with assistance from T.M. and H.‐B.P.; M.V.J., L.R.C., and B.K.W. analyzed the data; M.V.J. and R.A.F. led the writing of the manuscript. All authors contributed critically to the drafts and gave final approval for publication.

## Data Availability

Model data available from the Dryad Digital Repository: https://doi.org/10.5061/dryad.n89d4gg. Site‐level shorebird counts available from eBird (https://ebird.org/); URL links to all checklists in Supporting Information S9.
