# Supplementary material for: Multiple habitat use by declining migratory birds necessitates joined‐up conservation
Source: Ecol Evol. 2019 Feb 18;9(5):2505–15. doi: 10.1002/ece3.4895 (PMC6405493; doi:10.1002/ece3.4895)
Supplement: Supplementary file 1 [file ECE3-9-2505-s001.docx]

Jackson et al. Multiple habitat use by declining migratory birds necessitates joined-up conservation

Supplementary Material

**1 - Detailed description of survey sites**

From north to south, we carried out shorebird surveys at:

*Dongtai* (Figure 1B; approx. 32º45ʹ12ʺ N, 120º56ʹ60ʺ E): including the southern section of the seawall at Dongtai (~4.3km) and one of three large undeveloped ponds (~75ha total area). Observations were made from the seawall. Water cover on the pond was 30-50% over the three months of the survey period, with the rest of the pond containing bare mud interspersed with vegetation (vegetation cover 10-30%) which included *Phragmites australis* in the pond and largely herbaceous vegetation up to ~0.8m tall on the bunds surrounding the pond. We checked the two undeveloped ponds adjacent to the survey pond occasionally and they did not appear to provide suitable habitat for shorebirds due to high water cover. Ideally all three ponds would have been systematically surveyed but this was not feasible due to logistical constraints. The smaller ponds further inland were not accessible for surveys.

*Hai’an* (Figure 1C; approx. 32º40ʹ05ʺ N, 120º57ʹ13ʺ E): located just south of the Fangtang River, including a seawall (4.5km) and adjacent aquaculture pond complex (~600ha total area stretching approx. 2km inland from the intertidal flats; most individual ponds 2ha or smaller); surveys were conducted in the southern half of this area. To investigate whether any significant roosting occurred within the aquaculture complex, we conducted counts of randomly selected, accessible aquaculture ponds (determined primarily by track access and/or walking distance), stratified by distance from intertidal flats (< 1km and 1–2km from intertidal flats) and size (<3ha and >5ha). Nineteen randomly selected small ponds (< 3ha) and both of the larger ponds (> 5ha) in the survey area were surveyed.

*Fengli* (Figure 1D; approx. 32º31ʹ31ʺ N, 121º07ʹ05ʺ E): located just to the east of the Yangkou chemical factory zone, including a triangular-shaped aquaculture pond complex (20ha total; individual ponds 6ha or smaller) and an adjacent large undeveloped dry area (~50ha). Fengli was not originally selected as a survey area but was added to the survey schedule in October after shorebirds including Spoon-billed Sandpiper were observed aggregating there in September (L. Zhang pers obs). Eleven connected ponds of various shapes, sizes and condition forming an overall triangular shape were all counted, as well as one large, dry undeveloped pond (outlined in orange in Figure 1C).

*Ju Zhen* (Figure 1E; approx. 32º28ʹ19ʺ N, 121º13ʹ36ʺ E): located approximately 20km southeast of Yangkou town, including a seawall (4km) and adjacent aquaculture pond complex (~850ha total stretching 2km inland from the intertidal flats that included 4 large ponds immediately adjacent to intertidal flats (~150ha total area) and small ponds mostly 2ha or smaller) as well as a large claimed but currently undeveloped area immediately to the northwest of the intertidal flats (~380ha). The borders of the undeveloped pond comprised a seawall on three sides; the remaining side (furthest from the intertidal flats) was defined somewhat arbitrarily from a point at which heavy growth of *Spartina alterniflora* commenced and the entirety of the ground was thickly covered with *S. alterniflora*, forming a de facto edge to the pond. Water cover on the pond was 40-50% over the two months of the survey period, with the rest of the pond containing bare mud interspersed with *S. alterniflora*. To investigate whether any significant roosting occurs within the aquaculture complex, we conducted counts of randomly selected, accessible aquaculture ponds (determined primarily by track access and/or walking distance), stratified by distance from intertidal flats (< 1km and 1–2km from intertidal flats) and size (<2 ha and >10 ha). Sixteen randomly selected small ponds (< 2ha) and two of the four larger ponds (> 10ha) in the complex were surveyed.

*Dongling* (Figure 1F; approx. 32º19ʹ31ʺ N, 121º24ʹ58ʺ E): including approximately 1km of seawall and adjacent intertidal flats roosting area. Aquaculture ponds in the vicinity of the intertidal flats roost were scanned on numerous occasions but no evidence of artificial supratidal habitat use was observed.

**2 - Count schedule and results**

| Site | Count date | Tide condition* | Intertidal flat state | Total count all shorebirds |
| --- | --- | --- | --- | --- |
| Dongtai undeveloped ponds | 11/08/2017  17/08/2017  5/09/2017  7/09/2017  7/09/2017  16/09/2017  20/10/2017  20/10/2017 | High  High  High  Low  High  Low  Low  High | Covered  Uncovered  Uncovered  Uncovered  Covered  Uncovered  Uncovered  Covered | 21612  102  3300  2200  20100  97  1210  10890 |
| Hai’an intertidal flats roost | 12/08/2017  28/08/2017  12/09/2017  15/09/2017  18/10/2017  18/10/2017  21/10/2017 | High  High  High  High  High  High  High | Covered**  Covered**  Covered**  Uncovered  Covered**  Covered**  Covered** | 2419  8222  8411  5352  3657  3390  5175 |
| Hai’an aquaculture (number of ponds) | 26/07/2017 (1)  12/08/2017 (10)  12/08/2017 (10)  13/08/2017 (5)  13/08/2017 (5)  27/08/2017 (8)  27/08/2017 (2)  8/09/2017 (1)  8/09/2017 (3) 12/09/2017 (15)  15/09/2017 (2)  18/10/2017 (22)  21/10/2017 (1) | High  Low  High  Low  High  Low  High  Low  High  High  High  High  High | Covered  Uncovered  Covered  Uncovered  Covered  Uncovered  Covered  Uncovered  Covered  Covered  Uncovered  Covered  Covered | 334  762  4468  19  3247  13  3463  9  3658  628  0  1500  36 |
| Fengli (number of ponds) | 23/10/2017 (4)  24/10/2017 (12) | High  High | Covered  Covered | 4511  4165 |
| Ju Zhen undeveloped pond | 14/08/2017  16/08/2017  9/09/2017  9/09/2017 | High  High  High  Low | Covered  Covered  Covered  Uncovered | 5052  6627  3641  0 |
| Ju Zhen aquaculture (number of ponds) | 14/08/2017 (17)  14/08/2017 (15)  16/08/2017 (12)  9/09/2017 (16) | Low  High  High  High | Uncovered  Covered  Covered  Covered | 6  28  12  18 |
| Dongling intertidal flats roost | 10/08/2017  7/09/2017  21/09/2017 | High  High  High | Uncovered  Uncovered  Uncovered | 12338  15328  10831 |

*high = within three hours on either side of high tide; low = more than three hours from high tide

**count was completed immediately before the intertidal flats were covered, at which point we observed all birds depart from the intertidal flats

**3 – Maximum count of each shorebird species by location (number of ponds). Counts of international importance (> 1% of the flyway population) indicated in bold italics.**

|  | AUGUST |  |  |  | SEPTEMBER | |  | |  |  | OCTOBER |  |  | |
| --- | --- | --- | --- | --- | --- | --- | --- | --- | --- | --- | --- | --- | --- | --- |
| Location  (n ponds) | Dongtai (1) | Hai'an  (15) | Ju Zhen (1) | Ju Zhen (18) | Dongtai (1) | Hai'an (17) | Ju Zhen (1) | Ju Zhen (16) | | | Dongtai  (1) | Hai'an (21) | Fengli  (11) |  |
| Black-tailed Godwit | 300 | 606 | 0 | 0 | 0 | 0 | 0 | 0 | | | 0 | 0 | 0 | |
| Bar-tailed Godwit | 3000 | 182 | 0 | 0 | 0 | 0 | 5 | 0 | | | 0 | 0 | 0 | |
| Godwit sp | 0 | 0 | 230 | 0 | 0 | 0 | 0 | 0 | | | 0 | 0 | 0 | |
| Whimbrel | 2 | 5 | 29 | 0 | 0 | 1 | 0 | 0 | | | 0 | 0 | 0 | |
| Eurasian Curlew | ***2400*** | 1 | 0 | 0 | 0 | 0 | 0 | 0 | | | 590 | 8 | 0 | |
| Far Eastern Curlew | 5 | 1 | 0 | 0 | 0 | 0 | 0 | 0 | | | 0 | 7 | 0 | |
| Curlew sp | 0 | 0 | 14 | 0 | 1100 | 0 | 1 | 0 | | | 0 | 0 | 0 | |
| Spotted Redshank | 1 | 0 | 0 | 0 | 0 | 0 | 0 | 0 | | | 0 | 1 | ***485*** | |
| Common Redshank | 2 | 5 | 10 | 0 | 8 | 5 | 0 | 0 | | | 0 | 0 | 0 | |
| Marsh Sandpiper | 6 | 1 | 0 | 0 | 3 | 0 | 1 | 0 | | | 0 | 0 | 56 | |
| Common Greenshank | 60 | 9 | 40 | 4 | 18 | 6 | 2 | 4 | | | 19 | 60 | 39 | |
| Nordmann's Greenshank | ***250*** | 0 | 2 | 0 | 4 | 0 | 0 | 0 | | | 0 | 0 | 0 | |
| Green Sandpiper | 7 | 0 | 0 | 0 | 0 | 0 | 0 | 0 | | | 0 | 0 | 0 | |
| Wood Sandpiper | 0 | 0 | 0 | 0 | 0 | 0 | 0 | 0 | | | 0 | 0 | 1 | |
| Terek Sandpiper | 100 | 56 | 200 | 0 | 100 | 9 | 1 | 0 | | | 0 | 10 | 1 | |
| Common Sandpiper | 0 | 4 | 1 | 5 | 0 | 3 | 0 | 3 | | | 0 | 4 | 3 | |
| Grey-tailed Tattler | 1 | 3 | 0 | 0 | 0 | 3 | 12 | 1 | | | 0 | 0 | 0 | |
| Ruddy Turnstone | 100 | 52 | 280 | 8 | 0 | 11 | 0 | 2 | | | 0 | 0 | 0 | |
| Asian Dowitcher | 2 | 2 | 0 | 0 | 0 | 0 | 0 | 0 | | | 0 | 0 | 0 | |
| Great Knot | 4000 | 125 | 30 | 0 | 0 | 5 | 1 | 0 | | | 0 | 0 | 0 | |
| Red Knot | 300 | 5 | 0 | 0 | 0 | 1 | 0 | 0 | | | 0 | 0 | 0 | |
| Sanderling | 100 | 1 | 0 | 0 | 0 | 1 | 0 | 0 | | | 0 | 0 | 15 | |
| Red-necked Stint | 1000 | 160 | 200 | 14 | 11 | 42 | 370 | 0 | | | 0 | 0 | 28 | |
| Long-toed Stint | 0 | 0 | 0 | 0 | 0 | 2 | 0 | 0 | | | 0 | 0 | 0 | |
| Sharp-tailed Sandpiper | 5 | 732 | 30 | 0 | 0 | 42 | 5 | 0 | | | 0 | 0 | 0 | |
| Dunlin | ***6500*** | 2660 | 880 | 0 | 2 | 1093 | 1640 | 0 | | | 100 | 554 | 2909 | |
| Curlew Sandpiper | 0 | 2 | 0 | 0 | 0 | 0 | 0 | 0 | | | 0 | 0 | 0 | |
| Spoon-billed Sandpiper | 1 | 0 | 0 | 0 | 0 | 0 | 0 | 0 | | | 0 | 0 | ***20*** | |
| Broad-billed Sandpiper | 50 | 55 | 0 | 0 | 0 | 39 | 0 | 0 | | | 0 | 15 | 20 | |
| Far Eastern Oystercatcher | ***234*** | 3 | 0 | 0 | ***200*** | 0 | 3 | 0 | | | ***360*** | 0 | 0 | |
| Black-winged Stilt | 9 | 14 | 0 | 0 | 1 | 8 | 0 | 0 | | | 0 | 0 | 11 | |
| Pied Avocet | 17 | 0 | 0 | 0 | 200 | 0 | 0 | 0 | | | 0 | 0 | 11 | |
| Pacific Golden Plover | 0 | 0 | 0 | 0 | 0 | 0 | 0 | 0 | | | 0 | 0 | 1 | |
| Grey Plover | 1 | 10 | 102 | 0 | ***2000*** | 0 | 0 | 0 | | | ***1490*** | 0 | 0 | |
| Little Ringed Plover | 3 | 2 | 0 | 0 | 0 | 0 | 0 | 0 | | | 0 | 0 | 0 | |
| Kentish Plover | ***1600*** | ***1265*** | 0 | 5 | 16 | ***1119*** | 400 | 8 | | | ***1091*** | 826 | ***3181*** | |
| Lesser Sand Plover | 0 | 520 | 0 | 1 | 2 | 1621 | 0 | 0 | | | 0 | 50 | 78 | |
| Greater Sand Plover | 0 | 70 | 0 | 0 | 0 | 19 | 0 | 0 | | | 0 | 0 | 1 | |
| Sand Plover sp | 1600 | 280 | 0 | 0 | 0 | 0 | 800 | 0 | | | 0 | 0 | 0 | |
| Oriental Pratincole | 0 | 0 | 0 | 0 | 8 | 0 | 0 | 0 | | | 0 | 0 | 0 | |
| Unidentified small/medium | 0 | 300 | 4770 | 0 | 18000 | 200 | 400 | 0 | | | 8450 | 0 | 0 | |
| *TOTAL* | *21656* | *7131* | *6818* | *37* | *21673* | *4230* | *3641* | *18* | | | *12100* | *1535* | *6860* | |
|  | **AUGUST TOTAL** | |  | **35642** | **SEPTEMBER TOTAL** | | | **29562** | | | **OCTOBER TOTAL** | | **20495** | |

**4 – Mean total shorebird count from each supratidal pond**

| Survey region | Mean count (n = number of counts) ± SE; intertidal flats covered | Max number of species recorded; intertidal flats covered | Mean count (n = number of counts) ± SE; intertidal flats uncovered | Max number of  species recorded; intertidal flats covered |
| --- | --- | --- | --- | --- |
| Dongtai undeveloped large pond | **17534** (3) ± 3351 | 24 | **1382** (5) ± 619 | 12 |
| Hai’an intertidal flats roost | **5212** (6) ± 1046  (prior to intertidal flats being covered) | 20 | **5352** (1)  (birds remained on the intertidal flats for the duration of high tide) | 12 |
| Hai’an aquaculture complex* | **Individual ponds:**  HS1-12D: 1549 (3) ± 851  HS1-12W: 1459 (7) ± 670  HS1-3: 1(3) ± 0.6  HS1-5: 0 (3)  HS1-8: 1 (3) ± 0.9  HS2-1: 2 (3) ± 1  HS2-6: 0 (3)  HS2-8: 1 (3) ± 1  HS3-1: 0.5 (2) ± 0.5  HS3-4: 0 (2)  HS3-12: 512 (2) ± 144  HS3-18: 71 (2) ± 70  HS3-20: 45 (2) ± 7  HS4-4: 4 (2) ± 1  HS4-9: 9 (3) ± 8  HS4-13: 127 (2) ± 122  HS4-14: 179 (2) ± 153  HS4-21: 58 (3) ± 53  HS5: 126 (3) ± 105  HS6-4: 1 (2) ± 1  HS6-5: 1 (2) ± 0  HS6-6: 0(2)  **Total aquaculture area count**: 3355 (4) ± 641** | 17  19  1  0  3  2  0  3  1  0  6  7  3  2  3  3  3  2  7  1  1  0 | **Individual ponds:**  HS1-12D: 0 (1)  HS1-12W: 130 (6) ± 126  HS1-3: 0 (2)  HS1-5: 0 (2)  HS1-8: 0.5 (2) ± 0.5  HS2-1: 0 (2)  HS2-6: 0 (2)  HS2-8: 0 (2)  HS3-1: N/A  HS3-4: N/A  HS3-12: N/A  HS3-18: N/A  HS3-20: N/A  HS4-4: N/A  HS4-9: 3 (1)  HS4-13: 0 (1)  HS4-14: N/A  HS4-21: 0 (1)  HS5: 18 (1)  HS6-4: 0 (1)  HS6-5: 1 (1)  HS6-6: 0 (1)  **Total aquaculture area count**: 266 (3) ± 258** | 0  6  0  0  1  0  0  0  N/A  N/A  N/A  N/A  N/A  N/A  2  0  N/A  0  5  0  1  0 |
| Fengli aquaculture complex* | FE1: 429 (1)  FE2: 17 (1)  FE3: 149 (3) ± 54  FE4: 3668 (2) ± 29  FE5: 147 (2) ± 82  FE6: 5 (1)  FE7: 1 (1)  FE8: 0 (1)  FE9: 2 (1)  FE10: 0 (1)  FE11: 0 (1)  **Total aquaculture area count ***: 4810** | 2  4  3  10  8  2  1  0  1  0 | Not observed | N/A |
| Ju Zhen undeveloped large pond | **5107** (3) ± 862 | 16 | **0** (1) | 0 |
| Ju Zhen aquaculture complex* | YHS3: 0 (3)  YHS4: 0 (3)  YHS9-2: 11 (3) ± 6  YHS9-4: 3 (3) ± 1  YHS9-5: 2 (3) ± 2  YHS9-8: 1 (3) ± 0.6  YHS9-9: 1 (3) ± 0.3  YHS12-1: 0 (1)  YHS12-2: 0 (1)  YHS12-6: 0 (2)  YHS17-1: 0 (3)  YHS17-3: 0.7 (3) ± 0.3  YHS17-6: 0.7 (3) ± 0.3  YHS17-9: 0 (3)  YHS17-10: 0.3 (3) ± 0.3  YHS20-1: 0 (1)  YHS20-4: 0 (1)  YHS20-5: 0 (1)  **Total aquaculture area count**:**  **19 (3) ± 5** | 0  0  5  3  4  2  1  0  0  0  0  1  1  0  1  0  0  0 | YHS3: 0 (1)  YHS4: N/A  YHS9-2: 1 (1)  YHS9-4: 0 (1)  YHS9-5: 0 (1)  YHS9-8: 2 (1)  YHS9-9: 1 (1)  YHS12-1: 0 (1)  YHS12-2: 0 (1)  YHS12-6: 1 (1)  YHS17-1: 0 (1)  YHS17-3: 0 (1)  YHS17-6: 0 (1)  YHS17-9: 1 (1)  YHS17-10: 0 (1)  YHS20-1: 0 (1)  YHS20-4: 0 (1)  YHS20-5: 0 (1)  **Total aquaculture area count ***: 6** | 0  0  1  0  0  2  1  0  0  1  0  0  0  1  0  0  0  0 |
| Dongling intertidal flats roost | N/A – intertidal flats was never covered at this site | N/A | **12832** (3) ± 1322 | 22 |

*due to logistical constraints only a random sample of ponds from within these aquaculture complexes was surveyed so the total number of birds within the complex is expected to have been higher than the total observed in this study

** mean total aquaculture area count calculated using the maximum count for any ponds that were counted multiple times in one survey

*** total aquaculture area count calculated using the maximum count for any ponds that were counted multiple times in one survey; not a mean as this area was only surveyed once

**5 – Estimated number of days per month when the intertidal flats were covered in each survey region**

| Region | Tide height when intertidal flats were observed by the survey team to be covered | Tide chart used | Minimum number of days in 2017 where birds were required to use supratidal habitat  August September October | | |
| --- | --- | --- | --- | --- | --- |
| Dongtai | between 591 and 619cm | Jianggang | 11 | 12 | 10 |
| Hai’an | between 573 and 664cm | Yangkou | 17 | 18 | 17 |
| Fengli | unknown < 660cm | Yangkou | 18 | 18 | 17 |
| Ju Zhen | unknown < 589cm | Yangkou | 25 | 25 | 24 |
| Dongling | > 753cm* | Yangkou | 3 | 2 | 1 |

*at tide height 753cm the vast majority of birds were able to remain on the intertidal flats roost but the roost was very crowded and some birds (430 birds of 10831 total observed) left the roost and move to supratidal areas. It is therefore assumed that birds would have been pushed off the intertidal flats at tides > 753, but this was not actually observed.

**6 – Full model output of most supported model**

**Model: Shorebird abundance ~ Intertidal flats cover + Water cover + Vegetation cover + Bund + Size + Structures + (1 | Region) + (1 | Pond)**

| **Variable** | **Estimate** | **Std. Error** | **z value** | **Pr(>\|z\|)** |
| --- | --- | --- | --- | --- |
| Intercept | -1.71 | 1.25 | -1.37 | 0.17 |
| Intertidal flats cover | 2.61 | 0.50 | 5.20 | 2.02 e-07 *** |
| Water cover | -1.33 | 0.33 | 4.00 | -6.54 e-05 *** |
| Vegetation cover | 2.03 | 0.75 | 2.72 | 0.007 ** |
| Bund | -0.71 | 0.28 | -2.53 | 0.01* |
| Size | 1.12 | 0.31 | 3.63 | 0.0003*** |
| Structures | -1.21 | 0.41 | -2.95 | 0.003** |

Significance codes: 0 = ***; 0.001 = ** 0.01 = *

**7 - Detailed summary of count results and intertidal/supratidal dynamics at each site**

*Dongtai.* Very large aggregations of shorebirds were observed at high tide on the intertidal flats adjacent to the Dongtai seawall. We were unable to estimate numbers on the intertidal flats because birds occurred over a very large distance and the tide came in very quickly. However, on tides that covered the intertidal flats, almost all of the birds were observed crossing into the northernmost artificial supratidal pond shown in Figure 1B, which had a mean count of 14,307 ± 3,351 (n = 3; range 10,890 - 21,612) when the intertidal flats were covered and 1,382 ± 619 (n = 5; range 97 - 3,300) when the intertidal flats were uncovered. Birds were distributed in large groups throughout the dry areas of this pond. It was difficult to record shorebirds to species level within this pond because it is very large and could only be viewed from one side by standing on the seawall, so we were never able to record all birds to species level at this pond. We were nonetheless able to record a maximum of 24 shorebird species when the intertidal flats were covered compared with just 12 species over all counts when the intertidal flats were uncovered (Table 2 main paper). On the day (11 August 2017) when 24 species were recorded, Dunlin (~30%), Great Knot (19%), Bar-tailed Godwit (~14%) Eurasian Curlew (~11%), Kentish Plover (~7%) and Red-necked Stint (~5%) comprised almost 90% of all birds observed, though in later months Grey Plover also comprised a significant amount of the total (high count 2000 in October, ~11% of the total count). A minimum of 250 Nordmann’s Greenshank were observed during this count and this species was only observed in very small numbers at one other supratidal roost pond throughout the survey period, suggesting that this roost site is of particular importance to this species.

*Hai’an.* Shorebirds were observed aggregating at high tide on the intertidal flats adjacent to the aquaculture complex at Hai’an (Figure 1C). Mean count on the intertidal flats when they were later covered by the tide was 5,212 ± 1046 (n = 6) and 5,352 (n = 1) when the intertidal flats did not get covered by the tide (Table 2 main paper). On tides when the intertidal flats were covered, a significant number of shorebirds (generally small and medium sized) were observed flying inland from the seawall to roost within the aquaculture complex. However, the larger shorebird species were generally observed flying northward along the coast, possibly to join roosting flocks at Dongtai (located 8-10km north of the Hai’an intertidal flats and the closets known roost in the direction they were seen flying).

In August, large flocks of shorebirds (> 3,500) were observed on one large aquaculture pond adjacent to the seawall. When the water levels were low enough to expose significant banks and several islands within the pond, shorebirds roosted here and none were seen on the bunds. However, water levels in this pond were subsequently raised making it less suitable for shorebird roosting. Initially, many of the birds roosted on one of the bunds of this pond and in an adjacent very dry undeveloped pond. However by October both of these ponds has been largely abandoned by shorebirds, the former because of much higher water levels and the latter possibly as a result of disturbance (we observed dogs in this pond several times), and more birds were observed on smaller ponds throughout the rest of the aquaculture complex. In these smaller ponds, water cover was >95% on most ponds and most of the birds roosted on the bunds in between ponds, with some limited foraging on the narrow mud banks.

The average across all aquaculture ponds at Hai’an was 3355 ± 641 (n = 4) (Table 2 main paper). The maximum number of species recorded on the two adjacent aquaculture ponds that had many birds in August was 19, compared with a maximum number of 20 species observed on the intertidal flats. Nordmann’s Greenshank was observed on the intertidal flats but never in aquaculture ponds, and only very small numbers of Eurasian Curlew (max count on intertidal flats 435, max count on aquaculture ponds 8), Far Eastern Curlew (max count on intertidal flats 167, max count on aquaculture ponds 7) and Grey Plover (max count on intertidal flats 666, max count on aquaculture ponds 10) were seen on aquaculture ponds. Excepting the two aquaculture ponds discussed above that were mostly abandoned by shorebirds in October, the next highest number of species observed on any aquaculture pond was only 7 species. The species comprising the vast majority of individuals found in the aquaculture ponds at Hai’an were Dunlin, Kentish Plover, and Lesser Sand Plover.

*Fengli.* As this area was not originally selected to be surveyed, only one systematic survey of 11 ponds in a triangular-shaped aquaculture pond complex and an adjacent large dry area were carried out over two days, and only three ponds were surveyed more than once over the two days. A total of 4,810 birds (total aquaculture area count calculated using the maximum count for any ponds that were counted multiple times in the count period) was observed on these ponds but these were unevenly distributed. The dry undeveloped area (FE1) contained 429 (~10%) of the 4,469 birds observed and four recently drained aquaculture ponds (FE2-FE5) contained 4,032 (~90%) of the 4,469 birds observed (Supplemental Materials 4). Dunlin (~65%), Kentish Plover (24%), Spotted Redshank (~6%) and Lesser Sand Plover (~2%) comprised more than 96% of all birds observed and no larger bird species were present. The highest number of species observed on any one pond was 10 (Supplemental Materials 4). Twenty Spoon-billed Sandpipers were observed on one pond (FE3) and a minimum of 23 individual Spoon-billed Sandpipers were observed over two days (known because three individuals with leg flags seen on the first day were not seen subsequently on the second day), a huge total for the Yangkou area which has seen numbers of Spoon-billed Sandpipers decrease dramatically in recent years (L. Zhang pers obs). On the ponds that had shorebirds present in large numbers, water cover was significantly <100% and birds generally roosted or foraged in groups on exposed mud at the edges and in the centre of ponds. No birds were seen on bunds in this area.

*Ju Zhen.* Shorebirds were observed flying directly into the large undeveloped pond adjacent to the seawall at high tide in Ju Zhen (Figure 1E) without large aggregations of shorebirds being observed on the intertidal flats prior to entering this pond. Construction was occurring on the seawall that comprised the seaward boundary of this pond, but within the pond there was a significant amount of bare mud and shallow water, and little human activity. Shorebirds generally roosted or foraged in large groups in the middle of this pond some distance (>500m) inland from both the outer seawall and the wall adjoining it to the adjacent aquaculture complex. Mean shorebird count at this roost site when the intertidal flats was covered was 5107 ± 862 (n = 3) with a maximum of 18 species recorded when the intertidal flats were covered; no shorebirds were observed at the pond the one time we checked it when the intertidal flats were uncovered (Supplemental Materials 4). While not all birds could be identified to species level due to the distance from the observer to the pond, small birds dominated with Dunlin (24%), Sand Plover sp. (9%), Red-necked Stint (6%), Kentish Plover (4%), Ruddy Turnstone (3%), Terek Sandpiper (2%) and unidentified small/medium shorebirds (49%) comprising 97% of the total across two counts.

Very few shorebirds were observed within the aquaculture complex adjacent to this large undeveloped area. Of the 18 randomly selected ponds of varying size and distance from the intertidal flats surveyed, the highest mean count for any individual pond was only 11 birds (Supplementary Materials 4) and we did not observe any large flocks flying inland from the intertidal flats past the undeveloped pond roost. Water cover in these ponds generally approached 100% and birds were observed either on the bunds or on the very narrow muddy banks on the edge of ponds.

*Dongling.* Large aggregations of shorebirds were observed on the intertidal flats at Dongling (Figure 1F) with a mean count of 12,832 ± 1,322 (n=3). This was despite this area being heavily covered with *S. alterniflora* for 1-2km from the seawall out onto the intertidal flats. Even at very high tides, there was enough remaining intertidal flats around *S. alterniflora* patches that the birds could remain on the intertidal flats to roost. At a tide height of 753cm, some birds (430 birds of 10,831 total observed) did leave the roost and move to inland areas, so presumably this intertidal flats roost would have been covered at tide heights above 753cm. However, this only occurred 1-3 times per month during August, September and October 2017, so it is expected that most shorebirds used this intertidal flat roost and did not need to move to supratidal areas for the vast majority of the migration period.

**8 – Foraging results**

At the undeveloped pond at Dongtai, during the one count (7 September 2017) when we estimated foraging proportion when the intertidal flats were covered, <1% of the total number of sightings were birds observed foraging. During four counts in the pond when the intertidal flats were uncovered, across the 17 species observed, nearly half of the total number of sightings were birds observed foraging, including a high proportion of Lesser Sand Plover (100%), Little Ringed Plover (100%), Green Sandpiper (100%), Common Greenshank (69%), Red-necked Stint (65%), Kentish Plover (57%), Common Redshank (50%), unidentified shorebirds (50%), Marsh Sandpiper (44%), Pied Avocet (42%) and Black-winged Stilt (40%). Combined these results suggest that this pond was primarily used as a high tide roost but that there were some foraging opportunities.

*Table 8.1 Shorebirds observed foraging during counts in the large undeveloped pond at Dongtai when the intertidal flats were uncovered (n = 4; counts in August, September and October)*

| **Species** | **Total sightings** | **Number observed foraging** | **Proportion observed foraging** |
| --- | --- | --- | --- |
| Common Redshank | 10 | 5 | 0.5 |
| Marsh Sandpiper | 9 | 4 | 0.444 |
| Common Greenshank | 48 | 33 | 0.690 |
| Nordman's Greenshank | 4 | 0 | 0 |
| Green Sandpiper | 7 | 7 | 1 |
| Asian Dowicher | 1 | 0 | 0 |
| Red-necked Stint | 23 | 15 | 0.652 |
| Sharp-tailed Sandpiper | 1 | 1 | 1 |
| Dunlin | 102 | 3 | 0.029 |
| Far Eastern Oystercatcher | 200 | 0 | 0 |
| Black-winged Stilt | 10 | 4 | 0.4 |
| Pied Avocet | 41 | 17 | 0.415 |
| Grey Plover | 1 | 0 | 0 |
| Little Ringed Plover | 3 | 3 | 1 |
| Kentish Plover | 1139 | 650 | 0.571 |
| Lesser Sand Plover | 2 | 2 | 1 |
| Oriental Pratincole | 8 | 0 | 0 |
| unidentified small/medium | 2000 | 1000 | 0.5 |
| TOTAL | 3609 | 1744 | 0.483 |

At Hai’an, across the 29 species observed during 56 counts when the intertidal flats were covered, the only species where more than 2% of the total number of sightings were birds observed foraging were Black-winged Stilt (41%), Common Sandpiper (38%), Common Greenshank (13%), Red-necked Stint (5%) and Broad-billed Sandpiper (4%), and none of these occurred in large numbers. Only 1% of the total number of sightings were birds observed foraging. Combined, this indicates that the fish ponds at Hai’an, which generally had high water cover, were primarily used as high tide roosts.

*Table 8.2 Shorebirds observed foraging during counts at Hai’an when the intertidal flats were covered (n = 56; counts in August, September and October)*

| **Species** | **Total sightings** | **Number observed foraging** | **Proportion observed foraging** | **Number of counts seen** |
| --- | --- | --- | --- | --- |
| Black-tailed Godwit | 606 | 0 | 0 | 1 |
| Bar-tailed Godwit | 232 | 0 | 0 | 2 |
| Whimbrel | 7 | 0 | 0 | 3 |
| Eurasian Curlew | 10 | 0 | 0 | 3 |
| Far Eastern Curlew | 9 | 0 | 0 | 3 |
| Spotted Redshank | 1 | 1 | 1 | 1 |
| Common Redshank | 15 | 1 | 0.07 | 5 |
| Marsh Sandpiper | 1 | 0 | 0 | 1 |
| Common Greenshank | 67 | 9 | 0.134 | 13 |
| Terek Sandpiper | 116 | 0 | 0 | 8 |
| Common Sandpiper | 8 | 3 | 0.375 | 6 |
| Grey-tailed Tattler | 7 | 0 | 0 | 4 |
| Ruddy Turnstone | 95 | 0 | 0 | 5 |
| Asian Dowicher | 3 | 0 | 0 | 2 |
| Great Knot | 228 | 0 | 0 | 5 |
| Red Knot | 9 | 0 | 0 | 4 |
| Sanderling | 3 | 0 | 0 | 3 |
| Red-necked Stint | 312 | 16 | 0.051 | 8 |
| Long-toed Stint | 2 | 0 | 0 | 1 |
| Sharp-tailed Sandpiper | 1456 | 18 | 0.012 | 7 |
| Dunlin | 6867 | 88 | 0.013 | 14 |
| Curlew Sandpiper | 2 | 0 | 0 | 1 |
| Broad-billed Sandpiper | 137 | 6 | 0.044 | 10 |
| Far Eastern Oystercatcher | 5 | 0 | 0 | 2 |
| Black-winged Stilt | 17 | 7 | 0.411 | 7 |
| Grey Plover | 14 | 0 | 0 | 2 |
| Kentish Plover | 3626 | 14 | 0.004 | 21 |
| Lesser Sand Plover | 2273 | 0 | 0 | 7 |
| Greater Sand Plover | 92 | 0 | 0 | 4 |
| Sand Plover sp | 280 | 0 | 0 | 1 |
| unidentified small/medium | 500 | 0 | 0 | 1 |
| TOTAL | 17000 | 163 | 0.01 |  |

At Ju Zhen, feeding behaviour was recorded during two of the three counts when the intertidal flats were covered. Across the 20 species observed, species where more than 5% of the total number of sightings were birds observed foraging included Red-necked Stint (88%), Sharp-tailed Sandpiper (71%), Far Eastern Oystercatcher (67%), Common Redshank (50%), Common Greenshank (29%) and Grey-tailed Tattler (25%) (Table 5). About 7% of the total number of sightings were birds observed foraging. Combined, this indicates that this undeveloped pond was primarily used as a high tide roost with some opportunities for supplemental foraging for some species.

*Table 8.3 Shorebirds observed foraging during counts at Ju Zhen (large undeveloped pond) when the intertidal flats were covered (n = 2; counts in August and September)*

| Species | Total sightings | Number observed foraging | Proportion observed foraging |
| --- | --- | --- | --- |
| Bar-tailed Godwit | 5 | 0 | 0 |
| Godwit sp | 230 | 0 | 0 |
| Whimbrel | 22 | 0 | 0 |
| Curlew sp. | 1 | 0 | 0 |
| Common Redshank | 10 | 5 | 0.50 |
| Marsh Sandpiper | 1 | 1 | 1 |
| Common Greenshank | 42 | 12 | 0.286 |
| Nordmann's Greenshank | 2 | 0 | 0 |
| Terek Sandpiper | 201 | 1 | 0.005 |
| Common Sandpiper | 1 | 0 | 0 |
| Grey-tailed Tattler | 12 | 3 | 0.25 |
| Ruddy Turnstone | 280 | 0 | 0 |
| Great Knot | 31 | 0 | 0 |
| Red-necked Stint | 570 | 500 | 0.877 |
| Sharp-tailed Sandpiper | 35 | 25 | 0.714 |
| Dunlin | 2520 | 82 | 0.033 |
| Far Eastern Oystercatcher | 3 | 2 | 0.667 |
| Grey Plover | 102 | 0 | 0 |
| Kentish Plover | 400 | 20 | 0.05 |
| Sand Plover sp | 800 | 0 | 0 |
| unidentified small/medium | 5000 | 50 | 0.01 |
| TOTAL | 10268 | 701 | 0.06827 |

At Fengli, across the 17 species observed when the intertidal flats were covered, a significant proportion of the total number of sightings of Red-necked Stint (94%), Marsh Sandpiper (92%), Spoon-billed Sandpiper (86%), Black-winged Stilt (44%), Spotted Redshank (42%), Pied Avocet (36%), and Common Greenshank (14%) were birds observed foraging. Author LZ remained for several hours on both days at the pond where Spoon-billed Sandpipers were found and observed individuals feeding vigorously for extended periods of time and remaining in the pond to feed after the large group of Kentish Plovers also using the pond had departed for the intertidal flats after high tide. However, still only about 7% of the total number of sightings were birds observed foraging due to the large number of Dunlin and Kentish Plover not observed foraging (<1% and <3% of sightings, respectively). Overall this suggests that the partially drained fishponds at Fengli provided some substantive foraging opportunities for some species (including Spoon-billed Sandpiper) during late October, but were still used primarily as a high tide roost for the bulk of individuals observed at the site.

*Table 8.4 Shorebirds observed foraging during counts at Fengli when the intertidal flats were covered (n = 16; counts over two days in October)*

| Species | Total sightings | Number observed foraging | Proportion observed foraging |
| --- | --- | --- | --- |
| Spotted Redshank | 649 | 273 | 0.420647 |
| Marsh Sandpiper | 71 | 65 | 0.915493 |
| Common Greenshank | 57 | 8 | 0.140351 |
| Wood Sandpiper | 1 | 0 | 0 |
| Terek Sandpiper | 1 | 0 | 0 |
| Common Sandpiper | 3 | 0 | 0 |
| Sanderling | 17 | 2 | 0.117647 |
| Red-necked Stint | 49 | 46 | 0.938776 |
| Dunlin | 3814 | 29 | 0.007604 |
| Spoon-billed Sandpiper | 35 | 30 | 0.857143 |
| Broad-billed Sandpiper | 30 | 0 | 0 |
| Black-winged Stilt | 16 | 7 | 0.4375 |
| Pied Avocet | 11 | 4 | 0.363636 |
| Pacific Golden Plover | 1 | 0 | 0 |
| Kentish Plover | 3782 | 103 | 0.027234 |
| Lesser Sand Plover | 138 | 0 | 0 |
| Greater Sand Plover | 1 | 0 | 0 |
| TOTAL | 8676 | 567 | 0.065353 |

**9 – URLs for site-level shorebird counts submitted to eBird (**<https://ebird.org/>)

Hai'an aquaculture ponds 26/07/2017 <https://ebird.org/view/checklist/S50742475>

Dongling intertidal flats roost 10/08/2017 <https://ebird.org/view/checklist/S50742542>

Dongtai undeveloped pond 11/08/2017 <https://ebird.org/view/checklist/S50742584>

Hai'an aquaculture ponds 12/08/2017 <https://ebird.org/view/checklist/S50742600>

Hai'an intertidal flats roost 12/08/2017 <https://ebird.org/view/checklist/S50742654>

Hai'an aquaculture ponds 12/08/2017 <https://ebird.org/view/checklist/S50742626>

Hai'an aquaculture ponds 13/08/2017 <https://ebird.org/view/checklist/S50742772>

Hai'an aquaculture ponds 13/08/2017 <https://ebird.org/view/checklist/S50742828>

Ju Zhen aquaculture ponds 14/08/2017 <https://ebird.org/view/checklist/S50742861>

Ju Zhen undeveloped pond 14/08/2017 <https://ebird.org/view/checklist/S50742912>

Ju Zhen aquaculture ponds 14/08/2017 <https://ebird.org/view/checklist/S50742880>

Ju Zhen intertidal flats 14/08/2017 <https://ebird.org/view/checklist/S50742967>

Ju Zhen aquaculture ponds 16/08/2017 <https://ebird.org/view/checklist/S50742987>

Ju Zhen undeveloped pond 16/08/2017 <https://ebird.org/view/checklist/S50743016>

Dongtai undeveloped pond 17/08/2017 <https://ebird.org/view/checklist/S50743035>

Hai'an aquaculture ponds 27/08/2017 <https://ebird.org/view/checklist/S50743044>

Hai'an aquaculture ponds 27/08/2017 <https://ebird.org/view/checklist/S50743070>

Hai'an intertidal flats roost 28/08/2017 <https://ebird.org/view/checklist/S50743104>

Dongtai undeveloped pond 05/09/2017 <https://ebird.org/view/checklist/S50758782>

Dongtai undeveloped pond 07/09/2017 <https://ebird.org/view/checklist/S50758809>

Dongling intertidal flats roost 07/09/2017 <https://ebird.org/view/checklist/S50759049>

Dongtai undeveloped pond 07/09/2017 <https://ebird.org/view/checklist/S50758885>

Hai'an aquaculture ponds 08/09/2017 <https://ebird.org/view/checklist/S50759142>

Hai'an aquaculture ponds 08/09/2017 <https://ebird.org/view/checklist/S50759514>

Ju Zhen undeveloped pond 09/09/2017 <https://ebird.org/view/checklist/S50759649>

Ju Zhen aquaculture ponds 09/09/2017 <https://ebird.org/view/checklist/S50759819>

Ju Zhen undeveloped pond 09/09/2017 <https://ebird.org/view/checklist/S50759753>

Hai'an intertidal flats roost 12/09/2017 <https://ebird.org/view/checklist/S50760180>

Hai'an aquaculture ponds 12/09/2017 <https://ebird.org/view/checklist/S50759988>

Hai'an intertidal flats roost 15/09/2017 <https://ebird.org/view/checklist/S50760499>

Hai'an aquaculture ponds 15/09/2017 <https://ebird.org/view/checklist/S50760404>

Dongtai undeveloped pond 16/09/2017 <https://ebird.org/view/checklist/S50761925>

Dongling intertidal flats roost 21/09/2017 <https://ebird.org/view/checklist/S50762057>

Hai'an aquaculture ponds 18/10/2017 <https://ebird.org/view/checklist/S50762127>

Hai'an intertidal flats roost 18/10/2017 <https://ebird.org/view/checklist/S50762220>

Hai'an intertidal flats roost 18/10/2017 <https://ebird.org/view/checklist/S50762306>

Dongtai undeveloped pond 20/10/2017 <https://ebird.org/view/checklist/S50762375>

Dongtai undeveloped pond 20/10/2017 <https://ebird.org/view/checklist/S50762440>

Hai'an intertidal flats roost 21/10/2017 <https://ebird.org/view/checklist/S50762557>

Fengli aquaculture ponds 23/10/2017 <https://ebird.org/view/checklist/S50762720>

Fengli intertidal flats 23/10/2017 <https://ebird.org/view/checklist/S50762789>

Fengli aquaculture ponds 24/10/2017 <https://ebird.org/view/checklist/S50762871>
